# Droplet Digital Enzyme-Linked Oligonucleotide Hybridization Assay for Absolute RNA Quantification

**DOI:** 10.1038/srep13795

**Published:** 2015-09-03

**Authors:** Weihua Guan, Liben Chen, Tushar D. Rane, Tza-Huei Wang

**Affiliations:** 1 Department of Biomedical Engineering, Johns Hopkins University, Baltimore 21218, USA; 2 Department of Mechanical Engineering, Johns Hopkins University, Baltimore 21218, USA

## Abstract

We present a continuous-flow droplet-based digital Enzyme-Linked Oligonucleotide Hybridization Assay (droplet digital ELOHA) for sensitive detection and absolute quantification of RNA molecules. Droplet digital ELOHA incorporates direct hybridization and single enzyme reaction via the formation of single probe-RNA-probe (enzyme) complex on magnetic beads. It enables RNA detection without reverse transcription and PCR amplification processes. The magnetic beads are subsequently encapsulated into a large number of picoliter-sized droplets with enzyme substrates in a continuous-flow device. This device is capable of generating droplets at high-throughput. It also integrates in-line enzymatic incubation and detection of fluorescent products. Our droplet digital ELOHA is able to accurately quantify (differentiate 40% difference) as few as ~600 RNA molecules in a 1 mL sample (equivalent to 1 aM or lower) without molecular replication. The absolute quantification ability of droplet digital ELOHA is demonstrated with the analysis of clinical *Neisseria gonorrhoeae* 16S rRNA to show its potential value in real complex samples.

Precise and accurate quantification of biomolecules, such as gene copy number variations (CNV), DNA methylation level and gene expression, is essential for fundamental biomedical research as well as diagnostic applications^1^,^2^,^3^. To this end, various ‘digital’ assays (counting) have been developed to supplement traditional analog assays (signal integration). For example, digital polymerase chain reaction (PCR)^4^,^5^,^6^,^7^,^8^ and digital ELISA^9^ have been developed for absolute quantification of single DNA molecules and single protein molecules without the need for a standard curve. Although RNA (coding and non-coding) quantification is also highly desirable in many applications such as viral load tests^2^, RNA transcription expression, and microRNA profiling^10^, digital assays for absolute RNA quantifications remain less explored. The most prevalent RNA quantification method is based on reverse-transcription quantitative polymerase chain reaction (RT-qPCR), in which the reverse transcriptase first transforms RNA into complementary DNA (cDNA) before the cDNA is PCR amplified and monitored in real-time in order to perform post-amplification quantitative analysis. Although digital PCR is capable of enumerating rare RNA targets in one-step when combined with a reverse transcription process^11^,^12^,^13^,^14^, the nonlinear enzymatic reverse transcription process is known to introduce quantification bias^15^,^16^. In this regard, a non-reverse transcription based method may provide a better alternative for RNA quantification. Due to its single-stranded nature, linear hybridization is readily available for RNA molecules. While quantification in conventional hybridization assays (e.g., microarrays) is usually semi-quantitative and less sensitive, a digital version of hybridization based analysis can produce absolute quantification and ultra-high sensitivity simultaneously. For example, Walt and Duffy *et al*. have successfully implemented single enzyme based approaches for absolute quantification of bacterial genomic DNAs^17^. Pioneering works in single enzyme-based digital assays^18^,^19^,^20^ have led us to explore the possibility of using linear hybridization for digital RNA detection and quantification.

In this study, we report a continuous-flow droplet digital enzyme-linked oligonucleotide hybridization assay (droplet digital ELOHA) capable of absolute quantification of RNA molecules with high sensitivity. Droplet digital ELOHA incorporates direct hybridization and single enzyme reactions^18^,^19^,^20^, enabling a digital reverse-transcription-free RNA detection. Specifically, RNA targets are first allowed to hybridize to DNA capture probes that have been conjugated to magnetic beads. This is followed by hybridization to enzyme-labeled single-strand DNA detection probes. The magnetic beads are subsequently encapsulated into a large number of droplets, mixed with the enzyme substrate, incubated, and directly detected in a single integrated microfluidic chip. This enables facile digital assays without the need for material transfer between instruments. Furthermore, the continuous-flow operation removes the restriction of the total number of reactions imposed by the footprint of the device that is prevalent in micro-fabricated chamber-based digital assays.

## Methods

### Materials and Reagent

Surfactant poly(ethylene glycol) di-(krytox-FSH amide) was purchased from RAN Biotechnologies (MA, USA). Streptavidin coated magnetic beads (Dynabeads, diameter 1 μm), streptavidin-beta-galactosidase (SβG), resorufin beta-β-galactopyranoside (RGP), 2 M KCl, 1 M MgCl_2_, and ultraPure DNase/RNase-free distilled water were all purchased from Life Technologies. Purified synthetic target oligos, capture oligos and detection oligos (DNAs and RNAs) were custom manufactured by Integrated DNA Technologies. Bovine serum albumin (BSA) was purchased from Thermo Scientific. Sodium azide was from Sigma-Aldrich. 0.5 M EDTA (pH 8.0) was purchased from Corning Cellgro. Molecular biology grade Tween-20 (10%) was from Bio-Rad Laboratories. DEPC treated water and 1 M Tris-HCl (pH 8.0) was obtained from Quality Biological. Human total RNA was obtained from Promega. RNA extraction kit (miRNeasy Mini Kit) was from Qiagen.

### Design and Fabrication of Microfluidic Devices

A casting mold was fabricated using SU8–3050 photoresist and single layer soft lithography on 4 inch silicon wafer. The detailed layout of microfluidic design is presented in Supplementary Information (Figure S1). In our microfluidic devices, we used length-variable Tygon tubing to perform the incubation process in a continuous-flow fashion, which offers both manufacturing ease and incubation time flexibility as compared with designs that use several SU8 thicknesses for droplet generation and incubation^21^,^22^. The size of the nozzle for droplet generation is 10 μm and the height of the microfluidic chip is 20 μm. The microfluidic chips were made of polydimethylsiloxane (PDMS) by casting onto SU8 mold. The PDMS replica was permanently bonded with cover glass (130 μm thickness, Ted Pella) through oxygen plasma treatment. The microfluidic chips were further treated with Aquapel to render microfluidic channel surfaces hydrophobic before each use.

### Operation of Microfluidic Devices

A 50 μL solution of prepared magnetic beads and 50 μL RGP substrate solution were first introduced into Tygon tubes of diameter ~500 μm. This was then pushed by FC-40 oil using syringe pumps (Harvard Apparatus). We used a 60 μL/h pumping volumetric flow rate for the oil phase and 10 μL/h for both aqueous phases. The oil phase used in our experiments consists of FC-40 oil and 5% poly(ethylene glycol) di-(krytox-FSH amide) surfactant by weight. A 50 cm long Tygon tube (~102 μL in volume) was used for continuous incubation of droplets for about 77 min at room temperatures. The microfluidic chip was placed on a custom designed optical stage. The excitation laser beams were focused into the detection region on the chip using a 40× objective (Thorlabs RMS40X-PF, NA 0.75, focal depth~0.6 μm). The detection region is a 10 μm sized restriction which examines the incubated droplets one by one. Fluorescence data was continuously acquired from the detection region while upstream droplets were still being generated (Supplementary Movie). Running the assay at room temperature helped avoid droplet evaporation issues that may arise from thermal cycling on chip^8^. The droplets were imaged with a 4× objective before and after passing through the incubation tubing to verify the droplet stability.

### Optical Setup and Data Acquisition

**Figure 1:**
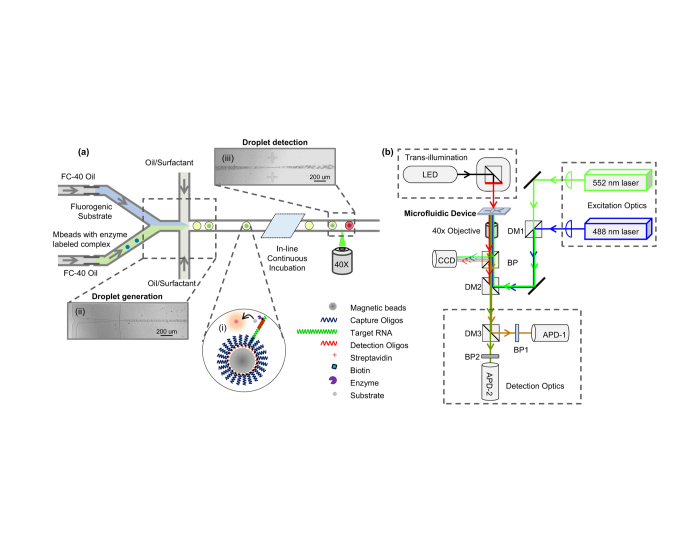
Continuous flow droplet digital ELOHA for absolute RNA quantification based on two dependent Poisson process. (**a**) Schematic of droplet digital ELOHA on a microfluidic chip. Initially, a sandwiched complex (inset i, first Poisson process) is formed by hybridization of oligos on magnetic beads. The enzyme labels (SβG) are then attached to the sandwiched complex through biotin-streptavidin interaction. This magnetic bead suspension and the fluorogenic substrate (RGP), each of a volume of 50 μL, are loaded into separate capillary tubes. Droplets are continuously generated by shearing the bead/substrate mixture with oil/surfactant on the device (inset ii, second dependent Poisson process). After continuous incubation of the droplets at room temperature in-line, the fluorescence intensity of each droplet is recorded one by one through a custom designed optical system (inset iii). (**b**) Schematic of custom designed optical system. The system includes a trans-illumination source for imaging the droplets on the chip, and two laser sources for fluorescence excitation. Abbreviation: DM, dichroic mirror; BP: band pass; APD: avalanche photodiode; CCD: CCD Camera.

The custom built optical setup for measuring droplet fluorescence is sketched in Fig. 1(b). The optical setup is capable of dual laser excitation (488 nm and 552 nm, OBIS lasers from Coherent, Inc.), as well as simultaneous dual color detection, including the ‘green’ (centered at 520 nm) and the ‘red’ (centered at 628 nm) wavelength bands. Laser power of 1 mW was used for excitation. The optical setup also has a trans-illumination LED source for imaging the droplets on the chip. A custom-built LabVIEW program was used to control fluorescent data acquisition using single photon counting module (SPCM-AQRH13) with 0.1 ms time bin.

### Data Analysis

A custom-built MATLAB (MathWorks) program was developed to analyze the data off-line. From the fluorescent intensity time trace, the program first finds the position of each droplet by searching the peaks of the data. Once each droplet is identified, the droplet size (Supplementary Figure S7) and intensity can be extracted by the program. The droplet size usually follows a Gaussian distribution. Due to a ‘digital’ nature of the assay, the detection is insensitive to the droplet size variation. A total amount of *N* (~1 million) droplets were analyzed for a specific experiment. The droplet intensity of these *N* droplets usually follows a bimodal distribution. The droplet populations with lower fluorescence intensity correspond to ‘negative’ reactions, where no single enzyme is present within these droplets. Populations with higher fluorescence intensity are ‘positive’ droplets which contain at least one enzyme label. The probability distribution *p*(*x*) of all droplet intensity is normalized so that 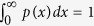. The percentage of positive droplets is calculated by 
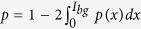
, where *I*_*bg*_ is the droplet background intensity that corresponds to the highest probability in the negative droplet population. This method greatly removes false positives from random changes in fluorescence during data acquisition.

### Single Enzyme Activity in Droplets

As-received SβG was mixed with 500 μL of 2 mM sodium azide to yield 2 mg/mL SβG stock solution, which was stored at 4 °C. Various SβG concentrations were prepared before each experiment by diluting the stock solution with the following buffer: 100 mM Tris-HCl (pH 8.0), 2 mM KCl, 1 mM MgCl_2_ and 0.2 mg/mL BSA. RGP substrate at the concentration of 500 μM was prepared in 100 mM Tris-HCl (pH 8.0), 2 mM KCl, and 1 mM MgCl_2_.

### Magnetic Beads Captured Single Enzyme Activity in Droplets

A total of 20 million streptavidin coated magnetic beads were resuspended into 20 μL of 2X binding and washing (BW) buffer (10 mM Tris-HCl (pH 8.0), 1 mM EDTA, 2 M NaCl, and 0.1% Tween-20), incubated with 3 pmol biotinylated capture oligos (5-/5BiotinTEG/GT TGT CAA GAT GCT ACC GTT CAG AG-3) for 30 min at room temperature. The beads were then washed 3 times with 1X BW buffer and resuspended into 20 μL of 1X BW buffer, which were further incubated with 7 pmol biotinylated complementary oligos (5-/5Biosg/CT CTG AAC GGT AGC ATC TTG ACA AC-3) for 3 hours at room temperature. Beads were then washed 3 times and made into 20 aliquots of 10 μL solutions (each had about 1 million magnetic beads). Each aliquot was incubated with SβG for 4 hours and washed 3 times.

### Mock-up ssDNA Quantification

Streptavidin coated magnetic beads were functionalized with biotinylated capture oligos (5-/5BiotinTEG/GT TGT CAA GAT GCT ACC GTT CAG AG-3). With excessive capture oligos removed, bead solution was used to capture mock-up ssDNA (5-TTG ACG GCG AAG ACC TGG ATG TAT TGC TCC TCT GAA CGG TAG CAT CTT GAC AAC-3) at various concentrations. After that, 1 pmol biotinylated detection oligos (5-TA CAT CCA GGT CTT CGC CGT CAA/3Bio/-3) were used to form a sandwich structure, from which SβG was tagged to the biotin site.

### RNA Quantification

#### Synthetic *Neisseria gonorrhoeae* 16S rRNA Target

Streptavidin coated magnetic beads were treated with DEPC and functionalized with biotinylated capture DNA probes (5-/5BiotinTEG/CGT TCG CCA CTC GCC ACC-3). Synthetic *Neisseria gonorrhoeae* 16S rRNA sample (5-GC AAG UCG GAC GGC AGC ACA GGG AAG CUU GCU UCU CGG GUG GCG AGU GGC GAA CG-3) were then captured by the beads, and then further hybridized with 1 pmol biotinylated detection DNA probe (5-GT GCT GCC GTC CGA CTT GC/3Bio/-3). SβG enzyme label was linked to the detection oligo through the biotin site.

#### Synthetic *Neisseria gonorrhoeae* 16S rRNA in Human Total RNA Background

In order to test the assay’s specificity towards RNA quantification in human total RNA background, we spiked clean 16S rRNA synthetic samples into 150 ng of human total RNA (equivalent to around 15,000 human cells).

#### Clinical *Neisseria gonorrhoeae* 16S rRNA from Isolated Cell Culture

A commercially available RNA extraction kit (miRNeasy Mini Kit, Qiagen) was utilized to extract RNA from bacterial cells, which were cultured from an isolation collected from a patient (Division of Infectious Diseases—Johns Hopkins Medicine). The concentration of eluted 80 μL of RNA sample was around 192.9 ng/μL (evaluated by Nanodrop). The protocols used to capture and label the *Gonorrhoeae* 16S rRNA were similar as those used in the synthetic targets.

### Real-time reverse transcription PCR for clinical Gonorrhoeae 16S rRNA

The unknown copy number of clinical *Gonorrhoeae* 16S rRNA was evaluated by real-time reverse transcription PCR. A forward primer (5- GCA AGT CGG ACG GCA GCA C-3) and reverse primer (5-CGT TCG CCA CTC GCC ACC-3) were used for reverse transcription and PCR. Reverse transcription was performed as follows: 20 μL of total volume consisting of 16 μL denatured RNA target, 1 × RT buffer, 40 units of murine RNase inhibitor and 10 units of Avian Myeloblastosis Virus reverse transcriptase. A modified real-time PCR protocol^23^ was performed using CFX96 system (Bio-Rad) as follows: 25 μL of total volume consisting of 1 μL cDNA template, 1 × PCR buffer, 1.5 mM MgCl_2_, 200 nM of each primer, 200 μM dNTP, 1 × EvaGreen, 0.8 M Betaine, 2 units Platinum *Taq* DNA polymerase and 14.4 μL ultrapure water. PCR products were also verified with melting curves, as well as electrophoresis in a 4% agarose gel.

## Results

### Two Steps of Dependent Poisson Process

Our sample preparation steps (forming enzyme labeled complexes on magnetic beads) share a similar procedure as that of digital ELISA^9^,^18^. As shown in inset i of Figure (a), RNA molecules were firstly captured and concentrated on magnetic beads through specific capture oligonucleotides, and then further hybridized with detection oligonucleotides labeled with enzyme reporters (SβG). The probability of finding *x* enzymes on a single magnetic bead is given by the Poisson statistics,


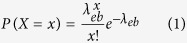


where *λ*_*eb*_ is the average enzyme per magnetic bead (equivalently, average RNA molecules per magnetic bead). When RNA molecules are much less than magnetic beads (e.g., *λ*_*eb*_ ~ 0.1), most magnetic beads (~99.5% for *λ*_*eb*_ ~ 0.1) will accommodate only 1 or 0 enzyme labeled complex.

After sample preparation step, these single enzyme labeled beads, together with fluorogenic substrate, were loaded into our microfluidic chip to generate digitalized droplets using a flow focusing method (inset ii of Fig. 1(a)). The probability of finding *y* beads in a single droplet also follows the Poisson statistics,


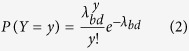


where *λ*_*bd*_ is the average number of magnetic beads in each droplet. If *λ*_*bd*_ ~ 1, Poisson statistics predicts more than 8% droplets contain more than 2 magnetic beads, which will greatly introduce the non-linearity for ‘digital’ counting of single molecules. In our experiment, 1 million magnetic beads in 50 μL of loading buffer were merged with another 50 μL of fluorogenic substrate to form droplets of size ~10 pL. As a result, *λ*_*bd*_ is around 0.1, which indicates that 90.48% droplets will contain no magnetic beads, 9.05% droplets will contain just one magnetic bead, and only 0.47% will contain more than 2 magnetic beads. In this regime, digital counting of single molecules is feasible. The next section details the quantification method for the aforementioned Poisson processes (Eqs (1) and (2)).

### Absolute Quantification Based on Two Dependent Poisson Processes

After incubation of magnetic beads with fluorogenic substrate, each droplet develops either a high or low concentration of florescent product, depending on whether the droplet contains enzyme reporter(s) (Fig. 1(a)). The droplet florescence intensity is measured by a custom designed optical system (Fig. 1(b)). We define those droplets without any enzyme reporters as *negative* droplets. There are several scenarios that yield negative droplets. For example, the droplet contains 0 beads, the probability of which is *P*(*Y *= 0), or the droplet contains 1 bead and this particular bead carries no enzyme, the probability of which is *P*(*Y *= 1)*P* (*X *= 0), or droplet contains 2 beads and both beads carries zero enzyme, the probability of which is *P*(*Y *= 2) *P*(*X *= 0) *P*(*X *= 0), and so forth. Therefore, the probability of all *negative* droplets is given by,


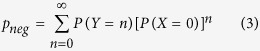


As a result, the probability of *positive* droplets is *p* = 1 − *p*_*neg*_. Substituting Eqs (1 and 2) into Eq. (3), the probability of *positive* droplets can be expressed as,


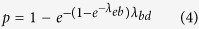


Note that for arbitrary *λ*_*ed*_ and *λ*_*bd*_, positive droplets can have many sophisticated combinations of beads and enzymes, including those with multiple beads and enzymes per droplet (e.g., two beads, one bead has 2 RNA linked enzymes and the other one has 1 RNA linked enzymes). For a linear ‘digital’ counting of RNA molecules in our continuous flow chip, we need both *λ*_*ed*_ and *λ*_*bd*_ to be small (~0.1) such that each single droplet will only contain 0 or 1 magnetic bead, and each single magnetic bead carries 0 or 1 enzyme.

To relate the number of captured RNA molecules with the measured percentage of positive droplets, we can derive the average RNA number per bead (equivalent to*λ*_*ed*_) from Eq. (4),


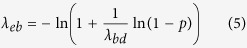


By knowing the total number of beads (*N*_*b*_, usually 10^6^ beads in our experiments), total testing sample volume loaded into the microfluidic chip (*v*_*s*_, 100 μL) and the droplet size (*v*_*d*_, 10 pL), the absolute number of *captured* RNA molecules is related to the percentage of positive droplets as,


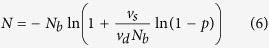


Since percentage of positive droplets (*p*) can be determined by examining the droplet florescence intensity one-by-one (inset iii of Fig. 1 (a)), absolute RNA quantification is possible through Eq. (6), with known experimental parameters of *N*_*b*_*, v*_*s*_, and *v*_*d*_. It is not required to know exactly which droplet contains a single magnetic bead. We believe this novel absolute quantification method can be broadly extended to other cases involving dependent Poisson processes, like double emulsions^24^.

### System Evaluation with ssDNA

**Figure 2:**
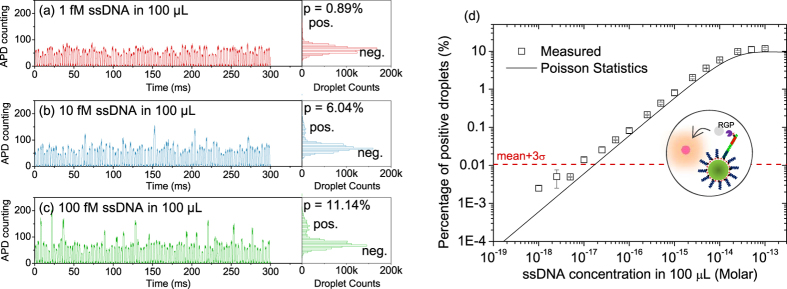
ssDNA quantification in 100 μL of samples. (**a**–**c**) Zoomed-in time traces of APD data and the histogram of the droplet florescence intensity for DNA concentrations of 1 fM, 10 fM, and 100 fM, respectively. A total amount of ~1 million droplets are interrogated in each experiment. A bin size of 5 is used for the histogram plot. (**d**) Percentage of the positive droplets plotted as a function of target DNA concentrations in 100 μL samples. The inset illustrates a magnetic bead capturing a single enzyme labeled complex and the corresponding enzymatic reaction. Dashed line is the background signal (mean plus 3 times of standard deviation). Error bars correspond to at least two measurements. Solid line is the Poisson statistic prediction using Eq. (4).

Since droplet digital ELOHA depends on the single enzyme activity in pL-sized droplets, we first verified that single enzyme activity is indeed functional and detectable in our droplet platform (Supplementary Text and Figures S2-S5). Before running absolute RNA quantification, we performed proof-of-concept experiments with synthetic single stranded DNA. As shown in Fig. 2(a–c), with increasing ssDNA concentration, more droplets have higher fluorescence intensity, indicating an increased population of droplets containing enzyme reporters. Figure 2(d) shows the change in the percentage of positive droplets as a function of ssDNA concentrations ranging from 100 fM to 1 aM.

The percentage of positive droplets is saturated at around 10% due to Poisson distribution of magnetic beads into droplets. If the average number of beads per droplet (*λ*_*bd*_) is ~0.1, ~90% of the droplets will contain no magnetic beads (Supplementary Figure S6). As a result, the maximal percentage of positive droplet is ~10%, no matter how many enzyme labeled complexes are associated with each magnetic bead during hybridization process. On the other hand, the false positive background, determined by experiments with no ssDNA target (mean+3σ), is found to be ~0.01% (dashed red line in Fig. 2). The saturation level of ~10% and the background level of ~0.01% sets a ~3 log linear range of our continuous flow droplet digital ELOHA based on two Poisson process.

The false positive background of ~0.01% is likely due to the non-specific binding of SβG enzyme onto the magnetic bead surface, which determines the limit of detection (LOD). The LOD of ssDNA quantification in 100 μL testing sample is around 10 aM, or 602 copies of ssDNA. Among different non-amplification based nucleic acid detection methods, the LOD of droplet digital ELOHA is comparable to SiMoA assay^17^, and better than methods that use gold nanoparticle scattering^25^, NanoString nCounter^26^ or imaging^27^ (see Supplementary Table 1 for comparison).

**Figure 3:**
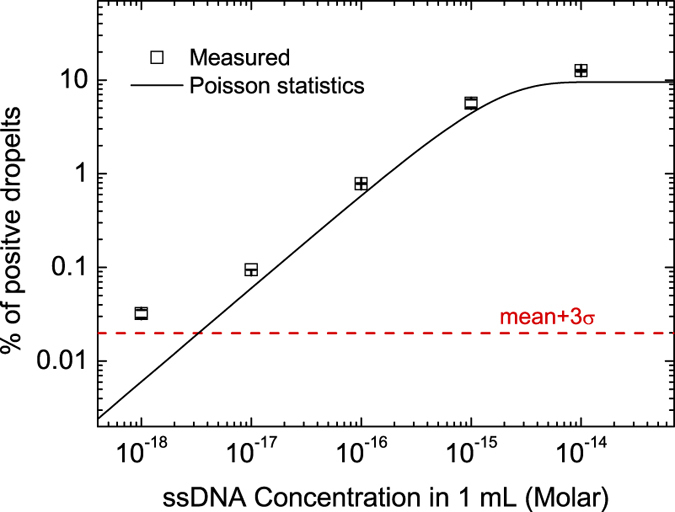
ssDNA quantification in 1 mL sample. Plot shows the percentage of the positive droplets as a function of target DNA concentrations in 1 mL of testing samples.

We also found the false positive background level remains almost the same when a 10 times larger volume of ssDNA is evaluated. Figure 3 shows the results for ssDNA in 1 mL volume, with LOD around 1 aM for 1 mL of ssDNA sample, or ~600 copies of ssDNA. This absolute detectable number (~600) is very close to that of the 100 μL sample (Fig. 2(d)), indicating that the background is mainly due to the enzyme non-specific binding to the magnetic beads. In addition, the ssDNA capture efficiency in 1 mL is very close to that in 100 μL sample when operating in the linear region (69 ± 7% for 100 μL sample and 63 ± 6% for 1 mL sample, see Supplementary Tables 2 and 3). The pre-concentrating effect of magnetic beads makes our droplet digital ELOHA an ultrasensitive tool for clinically relevant mL-sized sample volume^2^,^28^.

### Synthetic RNA Quantification

After system validation using ssDNA, we set out to explore our continuous flow droplet digital ELOHA for absolute RNA quantification without reverse transcription. *Neisseria gonorrhoeae*16S rRNA was used as a model RNA target. Similar protocols were used to form the enzyme labeled DNA-RNA-DNA complex on the magnetic beads, as in the case of ssDNA. Device operation was the same as before.

**Figure 4:**
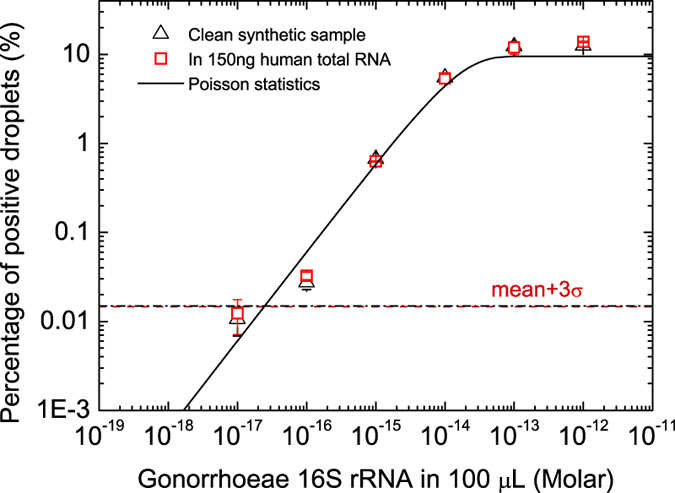
Quantification of a model RNA (synthetic *Neisseria gonorrhoeae* 16S rRNA). Plot shows the percentage of the positive droplets as a function of 16S rRNA concentrations in 100 μL of testing samples. The empty squares are results from clean synthetic sample, and the solid squares are results from samples with 150 ng of human total RNA background.

Figure 4 shows the results for clean synthetic 16S rRNA samples (triangles). The measured percentage of positive droplets agrees very well with the values predicted from the two-step Poisson statistics, indicating that the capturing and formation of the enzyme labeled complex is indeed functional with RNAs. No reference or standard is needed for quantification using Eq. (6). The false positive background is ~0.01%, attributed to the non-specific binding of enzyme to the magnetic beads.

To further verify the assay’s specificity towards the target RNA sequence, we spiked 16S rRNA sample into 150 ng of total human RNA background, which directly mimics the clinical test samples using an RNA extraction kit. The empty squares in Fig. 4 show the results from the spiked sample. It is found that the quantification of synthetic 16S rRNA is not affected by the background of 150 ng of human total RNAs (~15,000 copies of background molecules). The RNA capture efficiency by magnetic beads is found to be ~56 ± 1% for clean RNA sample and ~53 ± 5% for samples with total RNA background (see Supplementary Tables 4 and 5). The RNA capture efficiency is found to be less than the ssDNA case. The detection limit for both clean and spiked RNA sample is determined to be around ~600 molecules copies, an intrinsic background limit in our system mainly set by the sample preparation step (non-specific binding of enzymes).

### Clinical RNA Sample Quantification

Lastly, we applied our continuous flow chip for clinical RNA samples to evaluate its performance in real world situations. RNAs extracted from an isolation collected from patients were used as testing targets, which were specifically captured and hybridized on magnetic beads to form the enzyme labeled complex.

**Figure 5:**
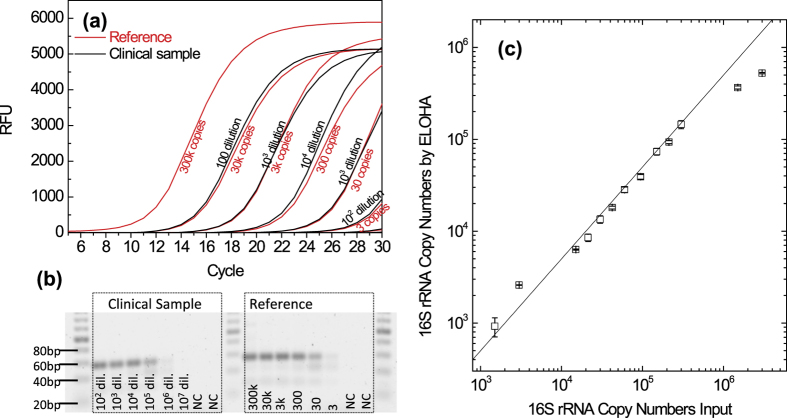
Clinical *gonorrhoeae* 16S rRNA quantification. (**a**) Real time reverse transcription PCR to determine the unknown 16S rRNA copy number in clinical samples. The synthetic sample is used as calibration reference standards. (**b**) Electrophoresis gel image shows that the amplified PCR product is the designed target DNA sequence with length of 54 bp. (**c**) 16S rRNA quantified by droplet digital ELOHA. Solid line is the eye-guiding line with a slope of 50%. Error bars correspond to triplicate measurements.

Before we ran RNA quantification using our droplet digital ELOHA, the copy number of *Gonorrhoeae* 16S rRNA was evaluated by real-time quantitative reverse transcription PCR (RT-qPCR). By using a reference standards (See Methods for details), the unknown starting 16S rRNA in 1 μL volume is found to be ~3 × 10^6^ copies (Fig. 5(a)). Gel-electrophoresis was adapted to verify the major amplification product is indeed the designed target (54 bp length, Fig. 5(b)).

Figure 5(c) shows the results from droplet digital ELOHA, where the RNA copy numbers were calculated through the measured value of positive percentage of droplets. It is found that the quantified number of RNA falls short of the expected value determined from the aforementioned RT-qPCR method. This is due to the fact that the RNA capture efficiency in ELOHA is not 100%. In fact, the RNA numbers determined by ELOHA are about half of the input copy number, which can be justified by the ~50% RNA capture efficiency (Supplementary Tables 4 and 5). Though sample-preparation induced quantification bias exists in almost any analytical assay, it is noteworthy that the RNA capture efficiency in ELOHA is fairly stable (~50%) across the linear dynamic range of the assay, therefore no calibration of the capture efficiency is required for each measurement. Currently we do not have a definitive conclusion to the ~50% capturing efficiency. We speculate this may due to the hybridization kinetics in low concentrations (digital assay region). In fact, a similar digital assay also reported a capture efficiency around 50–60%^9^. This is an interesting biophysical phenomenon worthy of further exploration, though it’s beyond the scope of this work. In addition, droplet digital ELOHA shows a consistently smaller coefficient of variation (<10%, Supplementary Figure S8) for triplicate and is able to reliably differentiate 40% difference in RNA quantity (Fig. 5(c)). This high accuracy of RNA quantification makes ELOHA very suitable for various applications such as absolute quantification of viral load^29^ and mRNA gene expression^30^.

## Discussion

By using magnetic beads to capture individual RNA molecules and digitally counting these molecules in a statistically significant quantity of droplets, digital ELOHA enables sensitive, specific and accurate quantification of RNA molecules without the need to generate cDNA using reverse transcription (as in digital RT-PCR), an enzymatic process that may introduce quantification bias. This makes digital ELOHA suitable for applications such as gene expression evaluation. Droplet digital ELOHA provides a novel method for a magnetic bead-based digital assay with a two-step Poisson process, which is particularly suitable for end-point detection based continuous-flow platforms, since no imaging is required. Our continuous-flow platform permits a small device footprint and a high throughput of analysis.

Though a very promising technology, some foreseeable limitations exist in the current platform and warrant further optimization. First of all, as with any hybridization based approach, the specificity may be compromised for long RNA strands which may result in false positive signals. On the other hand, for short RNA molecules (e.g., miRNA, ~22 nucleotides), the approach may suffer from low hybridization efficiency which will results in negative signals. These issues can be mitigated by using more specific probes^31^. Second, non-specific binding of enzyme reporters to magnetic beads should be suppressed for an improved dynamic range and detection limit. The current sample preparation results in a background of ~0.01%, which we believe can be further decreased by optimizing the surface block beyond the conventional BSA method. Third, the singleplex nature of the current platform is insufficient for multiplexed quantitative microRNA (miRNA) profiling^32^. Nevertheless, by incorporating multiple colors into our ‘cytometry’ system, it is a reasonable goal to achieve multiplexed detection in droplet digital ELOHA.

## Additional Information

**How to cite this article**: Guan, W. *et al*. Droplet Digital Enzyme-Linked Oligonucleotide Hybridization Assay for Absolute RNA Quantification. *Sci. Rep*. **5**, 13795; doi: 10.1038/srep13795 (2015).

## Supplementary Material

Supplementary Information

Supplementary Movie S1

## References

[b1] Bock C. Analysing and interpreting DNA methylation data. Nat. Rev. Genet. 13, 705–719, 10.1038/Nrg3273 (2012). 22986265

[b2] Ginocchio C. C. HIV-1 viral load testing—Methods and clinical applications. Lab. Med. 32, 142–152 (2001).

[b3] Cook E. H. & Scherer S. W. Copy-number variations associated with neuropsychiatric conditions. Nature 455, 919–923, 10.1038/Nature07458 (2008). 18923514

[b4] Vogelstein B. & Kinzler K. W. Digital PCR. Proc. Natl. Acad. Sci. USA 96, 9236–9241, 10.1073/pnas.96.16.9236 (1999). 10430926 PMC17763

[b5] Hindson B. J. . High-Throughput Droplet Digital PCR System for Absolute Quantitation of DNA Copy Number. Anal. Chem. 83, 8604–8610, 10.1021/Ac202028g (2011). 22035192 PMC3216358

[b6] Hatch A. C. . 1-Million droplet array with wide-field fluorescence imaging for digital PCR. Lab on a Chip 11, 3838–3845, 10.1039/C1lc20561g (2011). 21959960

[b7] Baker M. Digital PCR hits its stride. Nat. Methods 9, 541–544, 10.1038/Nmeth.2027 (2012).

[b8] Heyries K. A. . Megapixel digital PCR. Nat. Methods 8, 649–651, 10.1038/Nmeth.1640 (2011). 21725299

[b9] Rissin D. M. . Single-molecule enzyme-linked immunosorbent assay detects serum proteins at subfemtomolar concentrations. Nat. Biotechnol. 28, 595–599, 10.1038/Nbt.1641 (2010). 20495550 PMC2919230

[b10] Degliangeli F., Kshirsagar P., Brunetti V., Pompa P. P. & Fiammengo R. Absolute and Direct MicroRNA Quantification Using DNA-Gold Nanoparticle Probes. J. Am. Chem. Soc. 136, 2264–2267, 10.1021/Ja412152x (2014). 24491135

[b11] Takahashi K., Yan I. K., Kim C., Kim J. & Patel T. Analysis of extracellular RNA by digital PCR. Front. Oncol. 4, 129, 10.3389/fonc.2014.00129 (2014). 24926435 PMC4044516

[b12] Warren L., Bryder D., Weissman I. L. & Quake S. R. Transcription factor profiling in individual hematopoietic progenitors by digital RT-PCR. Proc. Natl. Acad. Sci. USA. 103, 17807–17812, 10.1073/pnas.0608512103 (2006). 17098862 PMC1693828

[b13] Pfitzner C. . Digital-Direct-RT-PCR: a sensitive and specific method for quantification of CTC in patients with cervical carcinoma. Sci. Rep. 4, 3970, 10.1038/srep03970 (2014). 24496006 PMC3913920

[b14] Racki N., Morisset D., Gutierrez-Aguirre I. & Ravnikar M. One-step RT-droplet digital PCR: a breakthrough in the quantification of waterborne RNA viruses. Anal. Bioanal. Chem. 406, 661–667, 10.1007/s00216-013-7476-y (2014). 24276251 PMC3892107

[b15] Sanders R., Mason D. J., Foy C. A. & Huggett J. F. Evaluation of digital PCR for absolute RNA quantification. PLoS One 8, e75296, 10.1371/journal.pone.0075296 (2013). 24073259 PMC3779174

[b16] Stahlberg A., Hakansson J., Xian X., Semb H. & Kubista M. Properties of the reverse transcription reaction in mRNA quantification. Clin. Chem. 50, 509–515, 10.1373/clinchem.2003.026161 (2004). 14726469

[b17] Song L. A. . Direct Detection of Bacterial Genomic DNA at Sub-Femtomolar Concentrations Using Single Molecule Arrays. Anal. Chem. 85, 1932–1939, 10.1021/Ac303426b (2013). 23331316

[b18] Shim J. U. . Ultrarapid Generation of Femtoliter Microfluidic Droplets for Single-Molecule-Counting Immunoassays. ACS Nano 7, 5955–5964, 10.1021/Nn401661d (2013). 23805985

[b19] Arayanarakool R., Shui L. L., Kengen S. W. M., van den Berg A. & Eijkel J. C. T. Single-enzyme analysis in a droplet-based micro- and nanofluidic system. Lab on a Chip 13, 1955–1962, 10.1039/C3lc41100a (2013). 23546540

[b20] Joensson H. N. . Detection and Analysis of Low-Abundance Cell-Surface Biomarkers Using Enzymatic Amplification in Microfluidic Droplets. Angewandte Chemie-International Edition 48, 2518–2521, 10.1002/anie.200804326 (2009). 19235824

[b21] Rane T. D., Chen L., Zec H. C. & Wang T. H. Microfluidic continuous flow digital loop-mediated isothermal amplification (LAMP). Lab Chip 15, 776–782, 10.1039/c4lc01158a (2015). 25431886 PMC4626017

[b22] Rane T. D., Zec H. C., Puleo C., Lee A. P. & Wang T. H. Droplet microfluidics for amplification-free genetic detection of single cells. Lab on a Chip 12, 3341–3347, 10.1039/C2lc40537g (2012). 22842841 PMC3696383

[b23] Henke W., Herdel K., Jung K., Schnorr D. & Loening S. A. Betaine improves the PCR amplification of GC-rich DNA sequences. Nucleic Acids Res. 25, 3957–3958, 10.1093/nar/25.19.3957 (1997). 9380524 PMC146979

[b24] Utada A. S. . Monodisperse double emulsions generated from a microcapillary device. Science 308, 537–541, 10.1126/science.1109164 (2005). 15845850

[b25] Storhoff J. J., Lucas A. D., Garimella V., Bao Y. P. & Muller U. R. Homogeneous detection of unamplified genomic DNA sequences based on colorimetric scatter of gold nanoparticle probes. Nat. Biotechnol. 22, 883–887, 10.1038/Nbt977 (2004). 15170215 PMC1201475

[b26] Geiss G. K. . Direct multiplexed measurement of gene expression with color-coded probe pairs. Nat. Biotechnol. 26, 317–325, 10.1038/Nbt1385 (2008). 18278033

[b27] Klamp T. . Highly Rapid Amplification-Free and Quantitative DNA Imaging Assay. Sci. Rep. 3, 1852, 10.1038/srep01852 (2013). 23677392 PMC3655336

[b28] Bettegowda C. . Detection of Circulating Tumor DNA in Early- and Late-Stage Human Malignancies. Sci. Transl. Med. 6, 224ra224, 10.1126/scitranslmed.3007094 (2014). PMC4017867 24553385

[b29] White R. A., Quake S. R. & Curr K. Digital PCR provides absolute quantitation of viral load for an occult RNA virus. J. Virol. Methods 179, 45–50, 10.1016/j.jviromet.2011.09.017 (2012). 21983150

[b30] Nolan T., Hands R. E. & Bustin S. A. Quantification of mRNA using real-time RT-PCR. Nat. Protoc. 1, 1559–1582, 10.1038/nprot.2006.236 (2006). 17406449

[b31] Zhang D. Y., Chen S. X. & Yin P. Optimizing the specificity of nucleic acid hybridization. Nat. Chem. 4, 208–214, 10.1038/nchem.1246 (2012). 22354435 PMC4238961

[b32] Pritchard C. C., Cheng H. H. & Tewari M. MicroRNA profiling: approaches and considerations. Nat. Rev. Genet. 13, 358–369, 10.1038/Nrg3198 (2012). 22510765 PMC4517822

