# Supplementary material for: Droplet Digital Enzyme-Linked Oligonucleotide Hybridization Assay for Absolute RNA Quantification
Source: Sci Rep. 2015 Sep 3;5:13795. doi: 10.1038/srep13795 (PMC4558716; doi:10.1038/srep13795)
Supplement: Supplementary Information [file srep13795-s1.pdf]

Droplet Digital Enzyme-Linked Oligonucleotide Hybridization Assay for Absolute RNA Quantification

Weihua Guan[1,2, 3,*], Liben Chen[2], Tushar D. Rane[1], Tza-Huei Wang[1,2,*]

[1]Department of Biomedical Engineering, Johns Hopkins University, Baltimore 21218, USA

[2]Department of Mechanical Engineering, Johns Hopkins University, Baltimore 21218, USA

[3]Present Affiliation: Department of Electrical Engineering, Pennsylvania State University, University Park 16802, USA

* Correspondence should be addressed to either *thwang@jhu.edu* or *w.guan@psu.edu*

Supplementary Text

Single Enzyme Activity in Droplets

Before performing droplet digital ELOHA experiment to quantify nucleic acid molecules, we carried out experiments to verify that single enzyme activity is indeed functional and detectable using our droplet microfluidic device and optical system. For this purpose, we loaded 50 µL of SβG solution of various concentrations and 50 µL of 500 µM RGP substrate into the microfluidic device. Droplets containing single SβG enzyme reporters and RGP substrate were continuously made, incubated and detected.

Supplementary Figure 2(a)-(c) show time traces of fluorescence data (only first 300 ms of data is shown) and the histogram of droplet fluorescence intensity from three representative concentrations of SβG tested. The bimodal distribution of droplet fluorescence intensity is clearly seen from the histogram plot. Increasing the concentration of SβG in the samples being tested results in the percentage of positive droplets from the droplet population to rise as expected. Supplementary Figure 2(d) shows the percentage of positive droplets as a function of all SβG concentrations tested. The measured values agree well with theoretical values predicted from Poisson statistics, which verifies single enzyme activity detection on our droplet system.

We also performed a negative control experiment with no SβG molecules (only buffer solution), the mean value and the standard deviation (σ) for the percentage of positive droplets is 0.01521% and 0.00227%, respectively. This negative control test sets the detection limit (3σ limit) to be 0.022%, equivalent to a SβG concentration of 36 aM in a 10 pL sized droplet. It is not possible to achieve this extremely low detection limit using standard bulk detection technology. To make a side by side comparison, we assayed 50 µL of SβG solution and 50 µL of 500 µM RGP substrate directly in a microtiter plate and measured the product fluorescence with typhoon 9410 imager (GE Healthcare). The detection limit for this bulk reaction is about 1.7 pM of SβG within the same amount of incubation time (Supplementary Figure 3). The droplet platform for single enzyme counting is thus 4-5 orders of magnitude more sensitive than the bulk measurement.

Magnetic Beads Captured Single Enzyme Activity in Droplets

After we confirmed detection of single enzyme activity in our droplet platform, we went on to use the magnetic beads to capture and concentrate SβG enzymes from the samples being tested. The objective of this test is (1) to verify the assay's compatibility with magnetic beads, and (2) to verify the pre-concentration role of magnetic beads by pulling down molecules of interest onto their surfaces. We used two biotinylated complementary DNA oligos to convert streptavidin coating of the beads into biotinylated surface so that these magnetic beads are able to capture streptavidin labeled SβG enzymes in 100 µL of sample. The captured SβG enzymes on the surfaces of 1 million beads are then eluted into 50 µL buffer and loaded into the microfluidic chip. The effective concentration of SβG in the loading sample is thus twice as high as in the original solution. It is noteworthy that we did not find aggregation of the magnetic beads during the course of the experiment. As a matter of fact, the magnetic beads are well separated into each droplet and their distribution follows a predicted Poisson distribution (Supplementary Figure 6).

Supplementary Figure 4 shows the percentage of positive droplets as a function of SβG concentration in the original 100 µL of testing sample. A dynamic range of over 4 orders of magnitude is achieved. The detection limit of SβG enzyme captured on the magnetic beads is about 20 aM, i.e., ~1000 enzyme molecules in 100 µL sample. In comparison, we also assayed the same reaction in a microtiter plate. The detected limit for the bulk measurement is about 850 fM of SβG within the same incubation time (Supplementary Figure 5).

Supplementary Figures

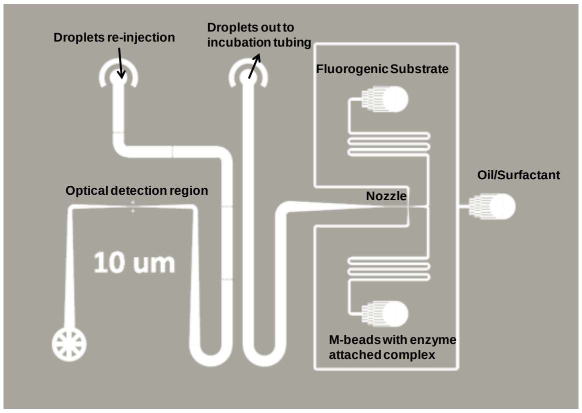

Supplementary Figure 1**.** Mask Layout for fabricating the device mold. In our microfluidic devices, we used length-variable tygon tubing to perform the incubation process in a continuous-flow fashion, which has overcome several difficulties in previous designs. First of all, the device fabrication is much easier than devices that use several SU8 thicknesses for droplet generation and incubation. Second, the reaction incubation time can be easily tuned by using tubing of different lengths, which offers more flexibility for different reactions. Third, the incubation region occupies less chip real-estate area. The manufacturing process becomes much more efficient (i.e., more devices in a single fabrication). Note that the passive filters near each injection port are designed to prevent the chip from clogging during the course of an experiment.

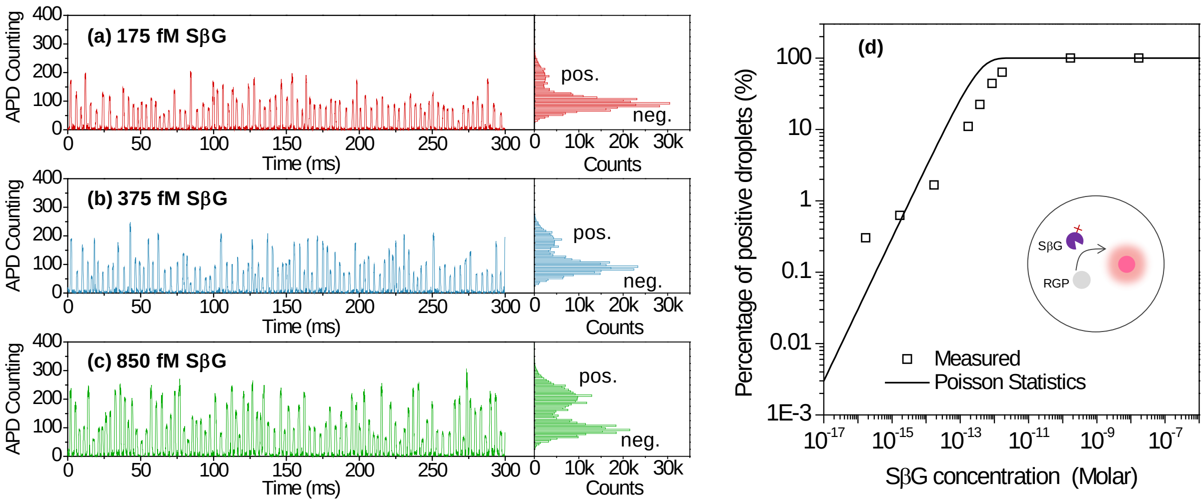

Supplementary Figure 2. Single enzyme activity in droplets. Zoomed-in time traces of APD data and the histogram of the droplet fluorescence intensity for (a) 175 fM SβG, (b) 375 fM SβG, and (c) 850 fM SβG. The bin size for the APD histogram plot is 5. (d) The percentage of the positive droplets as a function of SβG concentration in 50 μL testing sample. The inset illustrates the reaction in positive droplets. Solid line is the Poisson statistics prediction, $p = 1 - \exp(-CV_d N_A)$, where V_d is the droplet volume (10pL), and N_A is the Avogadro constant.

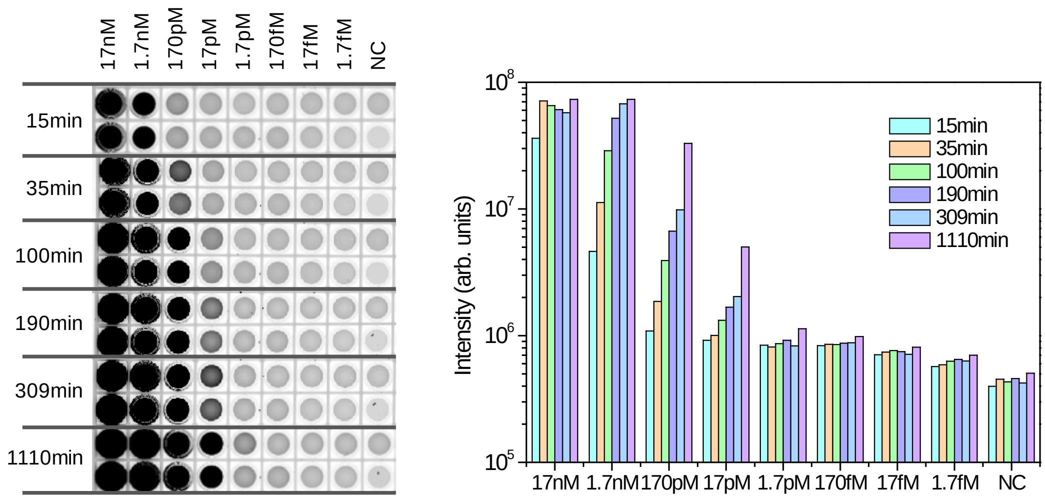

Supplementary Figure 3. Bulk reaction results of SβG with RGP. The detection limit is about

1.7 pM for the bulk measurement.

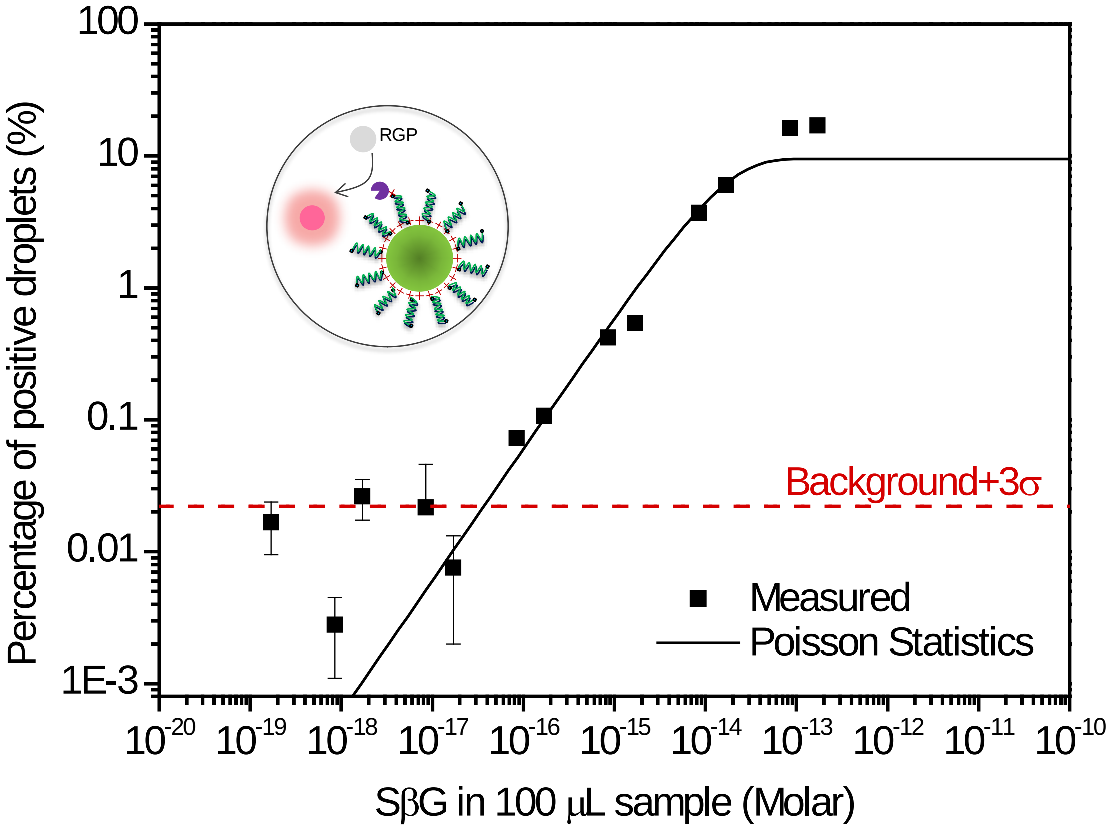

Supplementary Figure 4. Magnetic bead concentrated single enzyme activity in droplets. Plot shows the percentage of the positive droplets as a function of bulk SβG concentration in 100 μL sample (before magnetic bead capturing). The inset illustrates the corresponding reaction in positive droplets. Dashed line is the background signal (negative control sample) plus 3 times of standard deviation. The limit of detection (LOD) is about 20 aM. Error bars correspond to at least two measurements .

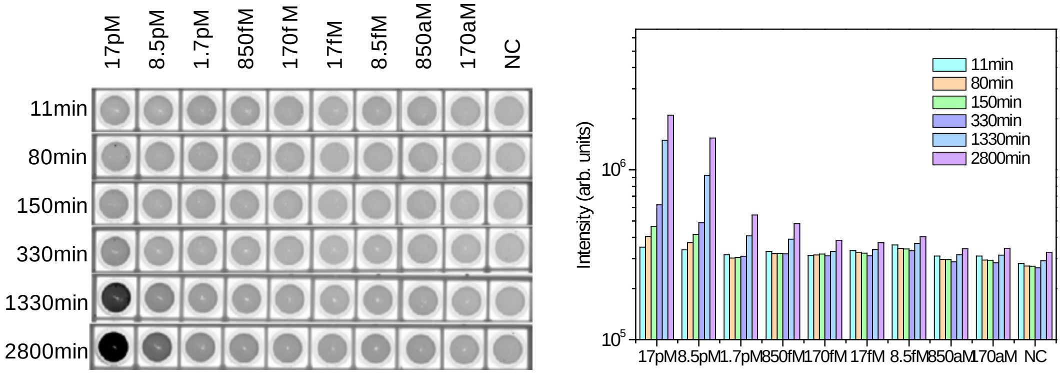

Supplementary Figure 5**.** The bulk reaction of concentrated SβG on beads with RGP. The detection limit is about 850 fM (for 100 µL test sample, using 1 million beads).

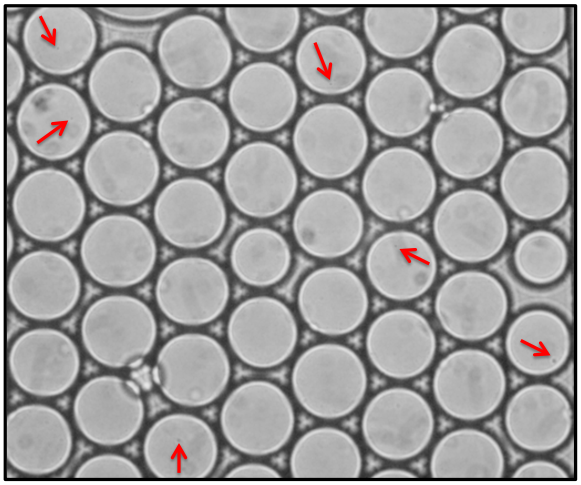

Supplementary Figure 6**.** Image of droplets with magnetic beads encapsulated, taken after the experiment. The magnetic beads are well separated into droplets. We observed 6 magnetic beads distributed into 48 droplets in the field of view, the average number of magnetic beads per droplet is 0.125, very close to the designed value of 0.1. Around 87.5% of droplets contain no magnetic beads.

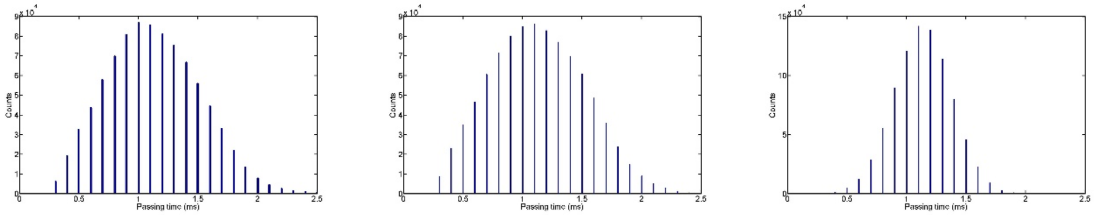

Supplementary Figure 7**.** Droplet size variation inter- and intra-experiments (3 randomly picked experiments). The time droplets pass the detection window is proportional to the droplet size.

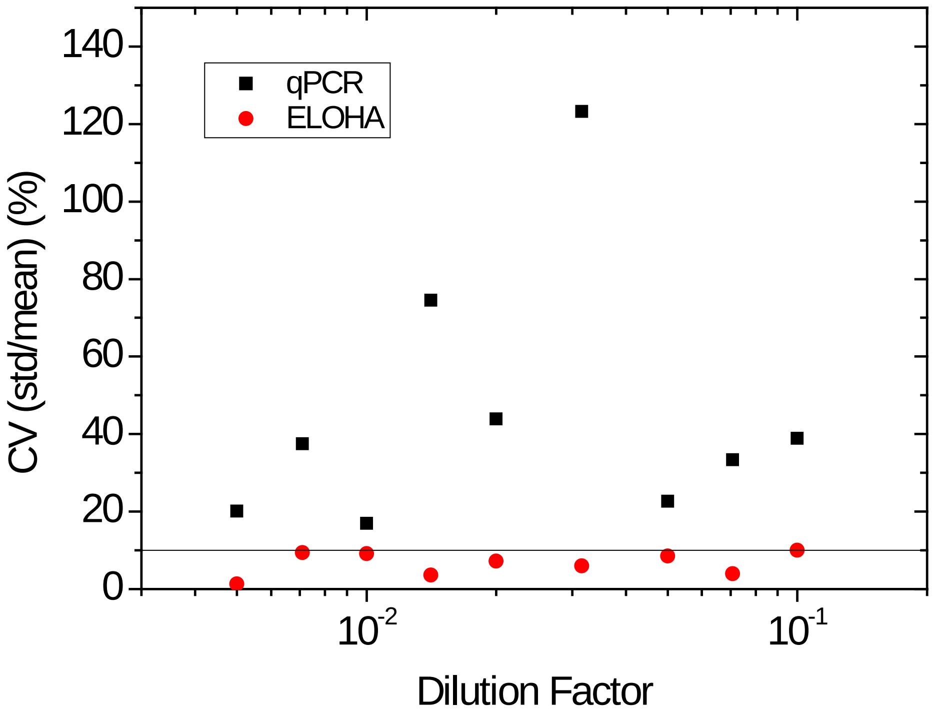

Supplementary Figure 8**.** Comparison of the coefficient of variation (CV) for qPCR and ELOHA quantification of clinical gonorrhoeae 16S rRNA sample.

Supplementary Tables

Supplementary Table 1. Comparison of the assay performances among different non-amplification based nucleic acid detection technologies

Method	LOD	Dynamic Range
Droplet digital ELOHA [this work]	~600 copies in 100 μL or 1 mL	1000
Branched DNA (1)	50 copies per 1 mL	NA
Gold nanoparticle scattering (2)	300,000 copies in a 4 μL	10,000
NanoString nCounter (3)	6000 copies in100 μL, multiplexing ability	500
SiMoA (4)	2100 copies in 50 μL	1000
Imaging Assay (5)	60,200,000 copies in 20 uL	NA

Supplementary Table 2. ssDNA in 100 µL sample

Concentration [M]	Droplets analyzed	Measured positive percentage	Measured λ_{eb} (Eq.5)	Measured # of captured molecules in 1 million beads	Measured # of captured molecules after background correction	Total # of enzymes in 100 µL sample	Capture efficiency
1E-14	959510	6.0421465%	0.3734133	373413	373234	602000	62%
1E-14	981575	5.7281410%	0.3494686	349469	349289	602000	58%
5E-15	970315	3.6124351%	0.2032980	203298	203119	301000	67%
5E-15	986319	3.5502713%	0.1993554	199355	199176	301000	66%
2.5E-15	971521	2.1063878%	0.1125468	112547	112368	150500	75%
2.5E-15	995348	1.9277680%	0.1023978	102398	102219	150500	68%
1E-15	975350	0.8915774%	0.0458123	45812	45633	60200	76%
1E-15	959916	0.7261052%	0.0371181	37118	36939	60200	61%
5E-16	976294	0.4135025%	0.0209356	20936	20757	30100	69%
5E-16	980586	0.4525865%	0.0229419	22942	22763	30100	76%
2.5E-16	974206	0.2082722%	0.0104792	10479	10300	15050	68%
2.5E-16	986984	0.2206723%	0.0111073	11107	10928	15050	73%
1E-16	971298	0.0732010%	0.0036681	3668	3489	6020	58%
1E-16	1004402	0.0898047%	0.0045024	4502	4323	6020	72%
5E-17	994911	0.0479440%	0.0024007	2401	2222	3010	74%
5E-17	1025190	0.0456501%	0.0022856	2286	2107	3010	70%
2.5E-17	1017624	0.0286943%	0.0014360	1436	1257	1505	84%
2.5E-17	980417	0.0223374%	0.0011176	1118	939	1505	62%

Supplementary Table 3. ssDNA in 1 mL sample

Concentration [M]	Droplets analyzed	Measured positive percentage	Measured λ_{eb} (Eq.5)	Measured # of captured molecules in 1 million beads	Measured # of captured molecules after background correction	Total # of enzymes in 1 mL sample	Capture efficiency
1E-15	860502	5.2601853%	0.3149563	314956	314190	602000	52%
1E-15	903296	6.0282565%	0.3723402	372340	371574	602000	62%
1E-16	884680	0.7877425%	0.0403461	40346	39579	60200	66%
1E-16	859652	0.7778729%	0.0398284	39828	39062	60200	65%
1E-17	820533	0.0942071%	0.0047237	4724	3957	6020	66%
1E-17	815550	0.0945374%	0.0047403	4740	3974	6020	66%

Supplementary Table 4. Clean Synthetic 16S rRNA in 100 µL sample

Concentration [M]	Droplets analyzed	Measured positive percentage	Measured λ_{eb} (Eq.5)	Measured # of captured molecules in 1 million beads	Measured # of captured molecules after background correction	Total # of enzymes in 100 µL sample	Capture efficiency
1E-14	851132	5.5022018%	0.3326352	332635	332300	602000	55%
1E-14	869151	5.4732722%	0.3305030	330503	330168	602000	55%
1E-15	876226	0.6768802%	0.0345491	34549	34214	60200	57%
1E-15	851020	0.6634392%	0.0338490	33849	33514	60200	56%

Supplementary Table 5. Synthetic 16S rRNA spiked in total Human RNA (100 µL volume)

Concentration [M]	Droplets analyzed	Measured positive percentage	Measured λ_{eb} (Eq.5)	Measured # of captured molecules in 1 million beads	Measured # of captured molecules after background correction	Total # of enzymes in 100 µL sample	Capture efficiency
1E-14	819100	5.8239531%	0.3567058	356706	356152	602000	59%
1E-14	817577	4.9046145%	0.2896155	289616	289062	602000	48%
1E-15	837889	0.6189364%	0.0315350	31535	30981	60200	51%
1E-15	822787	0.6345506%	0.0323462	32346	31793	60200	53%

Supplementary Table 6. Diffusion time (diffusion constant for nuclieic acid molecule is assumed to be around 1 $\mu m^2/s$). Due to its ability to capture and concentrate target molecules, droplet digital ELOHA is able to handle a large volume of testing samples. The consideration here is the time scale of mass transport as well as the binding kinetics. Thermodynamically, enough time should be allowed for target molecules to meet with magnetic beads with specific capture probes in a certain volume. As a result, a reasonable amount of incubation time is necessary during target molecule capturing.

Bead number	Sample Volume (μL)	Avg.Dist (μm)	Avg.Diff.Time(min)
1000000	100	46	35
10000000	100	22	8
1000000	1000	100	167
10000000	1000	46	35
1000000	10000	215	770
10000000	10000	100	167

Supplementary References

1. Collins ML, *et al.* (1997) A branched DNA signal amplification assay for quantification of nucleic acid targets below 100 molecules/ml. *Nucleic Acids Research* 25(15):2979-2984.
2. Storhoff JJ, Lucas AD, Garimella V, Bao YP, & Muller UR (2004) Homogeneous detection of unamplified genomic DNA sequences based on colorimetric scatter of gold nanoparticle probes. *Nature Biotechnology* 22(7):883-887.
3. Geiss GK, *et al.* (2008) Direct multiplexed measurement of gene expression with color-coded probe pairs. *Nature Biotechnology* 26(3):317-325.
4. Song LA, *et al.* (2013) Direct Detection of Bacterial Genomic DNA at Sub-Femtomolar Concentrations Using Single Molecule Arrays. *Analytical Chemistry* 85(3):1932-1939.
5. Klamp T, *et al.* (2013) Highly Rapid Amplification-Free and Quantitative DNA Imaging Assay. *Scientific Reports* 3:1852.
